# Acellular dermal matrix for one-stage treatment of lower extremity full-thickness skin defect: a case series

**DOI:** 10.1186/s12893-022-01871-x

**Published:** 2023-01-24

**Authors:** Gongchi Li, Qian Shen, Pan Zhou, Hanlin Liu, Jianghai Chen

**Affiliations:** 1 Department of Hand Surgery, Union Hospital, Tongji Medical College, Huazhong University of Science and Technology, 1277 Jiefang Avenue, Jianghan District, Wuhan, 430030 Hubei China; 2 School of Foreign Studies of Zhongnan University of Economics and Law, Wuhan, China

**Keywords:** Wound regeneration, Acellular dermal matrix, One-stage repair

## Abstract

**Background:**

Self-repair of lower limb wounds has always been one of the research hotspots. Flaps and skin graft are the preferred treatment for lower extremity wound reconstruction. However, these treatments have many disadvantages, such as secondary damage, poor healing quality. In recent years, the use of acellular dermal matrix has emerged as an alternative treatment option for extremity ulcers.

**Methods:**

This study aimed to explore whether acellular dermal matrix can be used as a single treatment to promote wound healing. 7 patients with lower extremities cutaneous deficiency exposing bone or tendon, were covered by Pelnac, which was an acellular dermal matrix product approved by China Food and Drug Administration. All the wound was treated by Pelnac without flaps and skin graft. The external dressing was changed every 10 days.

**Results:**

After a maximum of 20 weeks, all the wounds were completely healed. During the 12 months follow-up period none of the patients developed skin wear on the treatment area. All patients maintained their postoperative ambulatory ability. All patients were satisfied with the appearance and feeling after wound healing.

**Conclusion:**

These findings may mean acellular dermal matrix is a novel method offering opportunity for treatment of lower extremities cutaneous deficiency exposing bone or tendon. It also has the potential to close wounds of all uninfected, non-ischemic, full-thickness cutaneous deficiency.

## Introduction

The skin is the largest organ covering the human body. Continuous loss of normal anatomic structures and function of the skin results in a wound [1, 2]. Based on their wound healing time frames, wounds are classified as acute or chronic. In everyday pathology, wounds remain a challenging clinical problem, with early and late complications representing a frequent cause of morbidity and mortality [3, 4]. Acute wounds constitute a common health problem, with an estimated 11.8 million wounds treated in emergency departments in the USA annually [5, 6]. Wounds are typically characterized based on wound depth and the area of skin affected [7]. According to the degree of skin injury, wounds can be divided into partial-thickness wounds and full-thickness wounds [8]. There are numerous therapeutic methods used to treat different types of wounds, such as negative pressure wound therapy, bioengineered tissue alternatives, pedicle flaps, and free tissue transfer. Based on the “reconstruction pyramid”, which is a guide for wound healing, treatments are arranged hierarchically, from minimally invasive procedures for small and superficial wounds to highly complex procedures for large, deep, and complex soft tissue defects [9]. For instance, due to a lack of redundant or pliable surrounding soft tissue, wounds in the foot or ankle often need to be closed with various kinds of flaps. However, surgical flap interventions have associated disadvantages that include secondary damage, poor tissue appearance, and high surgical requirements. For this reason, tissue engineered dressings have better application prospects than flap surgery. Acellular dermal matrix (ADM) is a promising tool for the treatment of soft tissue defects that can maintain an enhanced quality of wound repair [10–12]. In recent years, acellular dermal matrix (ADM) has been used in the treatment of deep tissue defects in combination with split-thickness skin grafts and negative pressure wound therapy [13, 14]. However, ADM also has the disadvantage that it cannot independently achieve physiological epithelial tissue regeneration and repair [15]. Here, we found that a porcine acellular dermal matrix can promote complete wound healing.

## Materials and methods

All patients provided informed consent to participate in the study and for the use of their images. Inclusion criteria: full-thickness skin defect below the knee joint. Exclusion criteria: Patients with diabetes mellitus, lower extremity vascular disease or osteomyelitis. We used Pelnac (Gunze Corp., Kyoto, Japan) (an artificial dermal product approved by the China Food and Drug Administration) for wound treatment. The Pelnac double layer is composed of an atelocollagen sponge layer and a silicone layer.

Seven patients with lower extremity cutaneous deficiency with exposed bone or tendon were treated in our department from December 2014 to January 2017. This prospective study included a 12-month follow-up, with an overall study period of 3 years. Patient age ranged between 8 and 55 years (mean age 40.6 years). None of the patients had concomitant flap or fascia flap surgery, and none presented with vascular disease or diabetes. Postoperative sensory status was evaluated through two-point discrimination measurements and assessments of total active motion.

All surgical procedures were performed under epidural anesthesia with the patient in a supine position. A tourniquet was placed around the lower limb to limit intraoperative bleeding. After debridement and hemostasis, the wound was rinsed three times with H2O2 and 0.1% povidone iodine. Afterwards, the wound was covered with Pelnac according to the manufacturer’s protocol. Subsequently, the Pelnac was trimmed to precisely contour the wound and then immersed in saline for 15 s before application. The Pelnac was sutured to the defect using 5/0 Prolene sutures. To facilitate wound exudation, small drainage holes were made in the silicone film. After the operation, gauze and bandages were applied to cover the surface of the Pelnac. The dressings were changed every 2–5 days depending on the exudation, and outpatient or online visits were performed every 2–3 weeks. The silicone film was only detached from the wound after complete healing.

### Statistical analysis

Statistical analysis consisted of the comparison between the affected side and healthy side in terms of two-point discrimination measurements and total active motion, performed using the Wilcoxon signed-rank test. The level of statistical significance was set at p < 0.05.

## Results

In this study, 7 patients (age range 6–62 years) were treated with Pelnac for lower extremity wounds with bone or tendon exposure (wound size range 5–49.5 cm^2^). All wounds resulted from tissue removal and debridement, and defects were filled by suturing the Pelnac in place. Final wound healing occurred within 13 weeks on average (range 7–20 weeks). One or two linear scars [16] were left in the original wound area, depending on wound shape. The width of the scars varied from 1 to 15 mm depending on the initial size of the wounds.

**Fig. 1:**
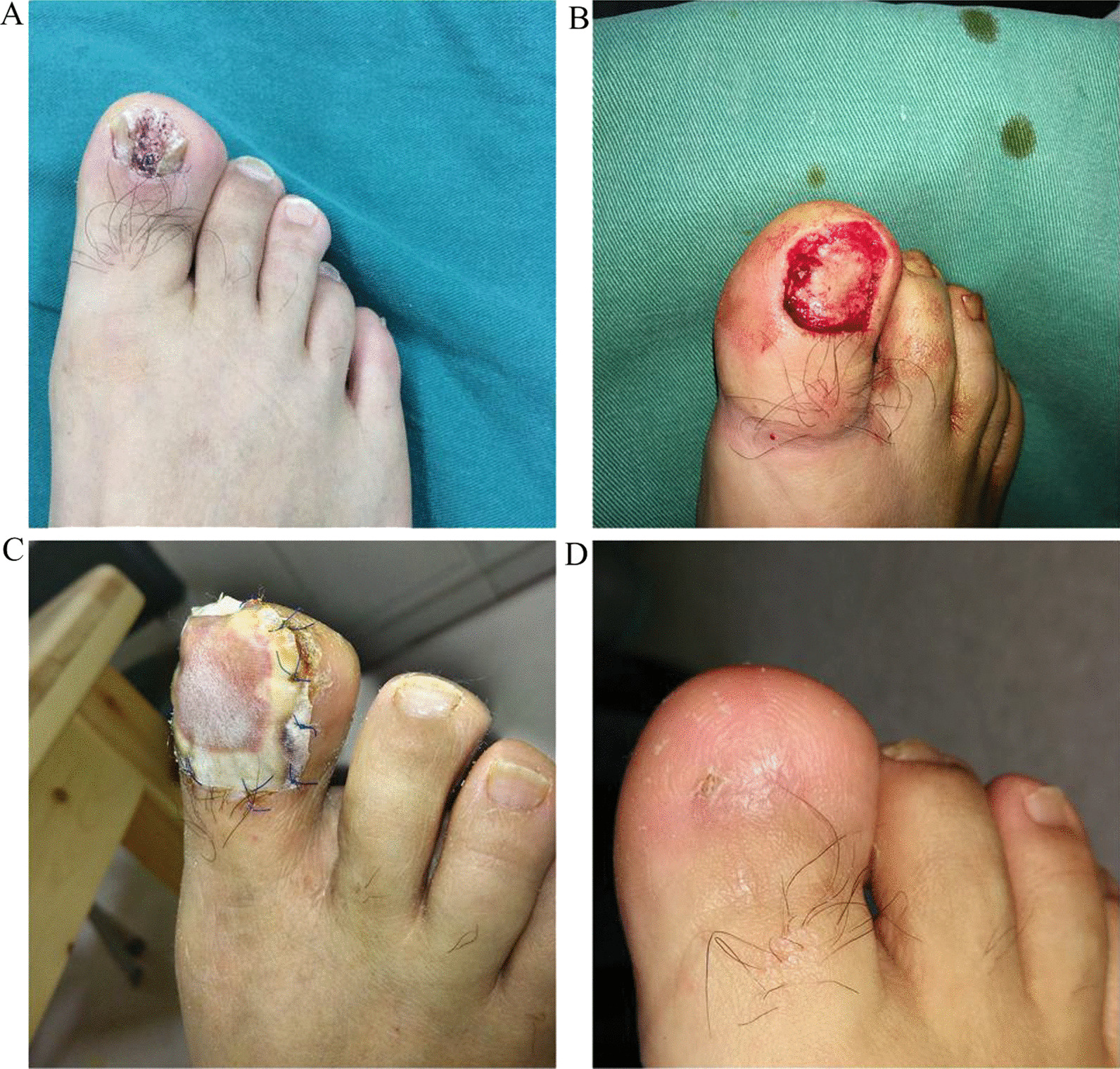
Case 1 (41-year-old male): **A** Big toe chronic fungal infection. **B** after resection of the lesion, an armor bed defect with last phalanx exposure. **C** the wound was covered with Pelnac. Wound healed for 22 weeks. **D** After half a year of wound healing, there was only one small scar and a new skin texture

**Fig. 2:**
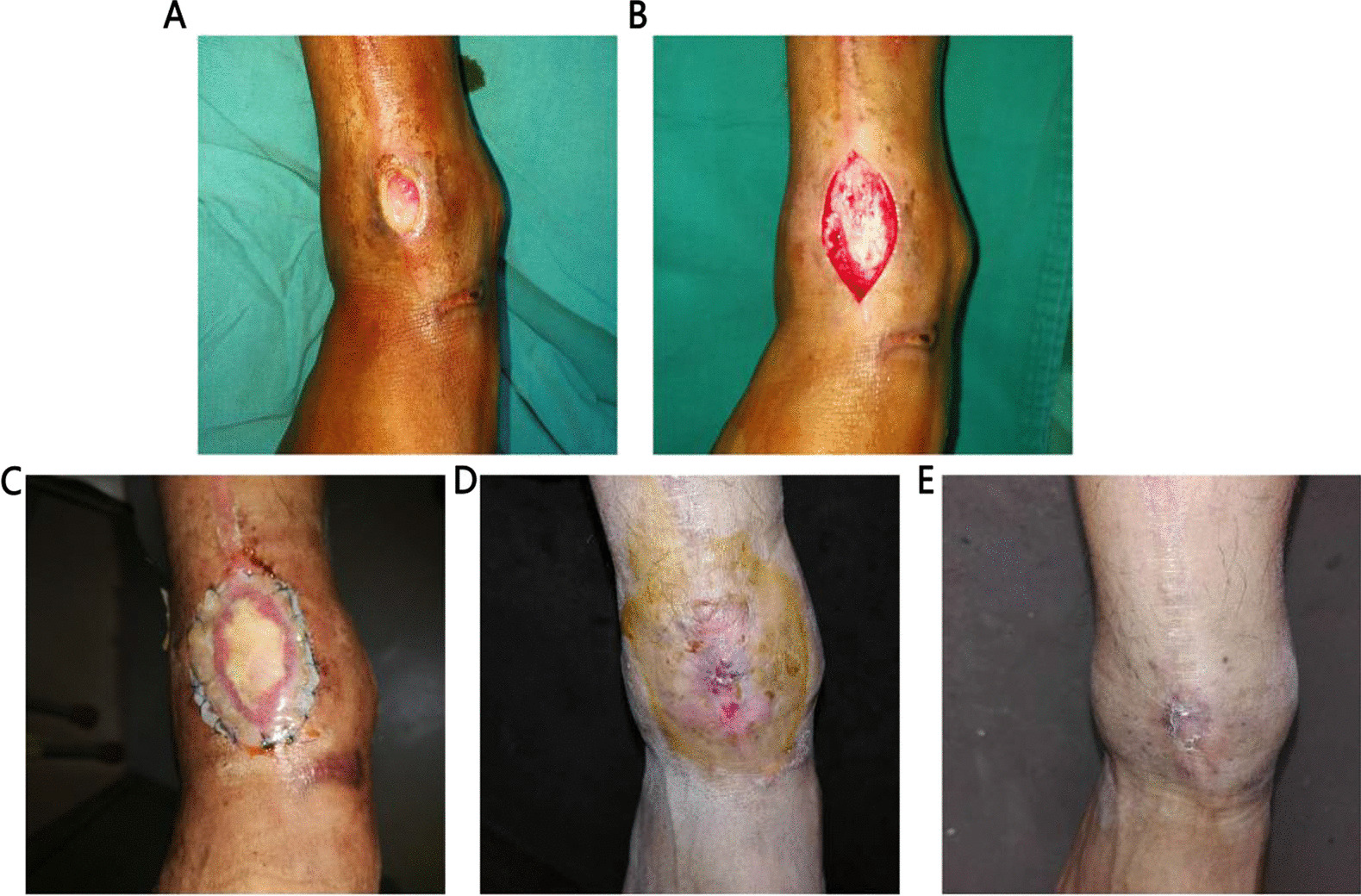
Case 2 (35-year-old male): **A** Chronic ulcer in front of the ankle. **B** Skin defect after enlarged excision. **C** the wound was covered with Pelnac. **D** 3 months post-operation. **E** 8 months after surgery, wound healed with a small “O” type linear scar

**Fig. 3:**
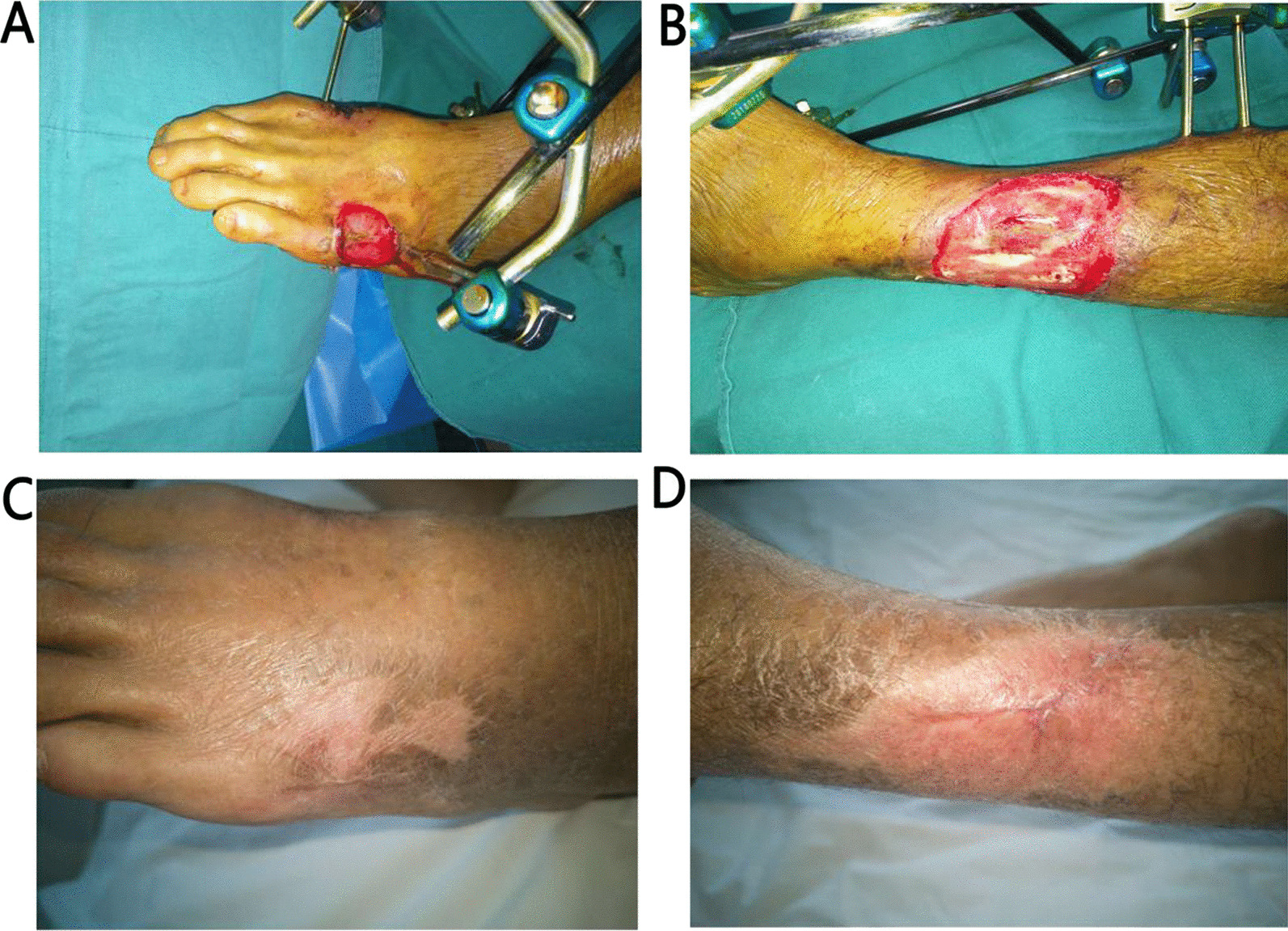
Case 3 (66-year-old male): **A**, **B** electric burn in left lower extremity. Skin defects with bone and tendon exposure were observed after debridement. **C**, **D** After treatment with ADM, wound healed within 18 weeks. After 14 months, there was only one small scar and a new skin texture

After 12 months of follow-up, skin sensation and joint movement were evaluated in all patients. All patients achieved a return to normal sensation, although slight variations in sensation between the healthy side and the affected side were noted in a few instances. At the one-year follow-up, the healed wound was free of significant pain, and the range of joint motion averaged 90% of that of the healthy side (Table 1). None of the patients showed signs of skin damage or trouble walking. All patients returned to normal daily life. Specific cases can be seen in Figs. 1, 2, 3.

**Table 1:** Sensation, pain and movement after wound healing

No	Site of wound	2-PD (mm)		VAS	TAM (%)
		Uninjured side	Injured side		
1	Front of ankle	13.2	15.2	2	90
2	Achilles tendon area	15.7	20.8	3	93
3	Big toe	5.4	7.3	1	94
4	Achilles tendon area	14.7	19.2	2	85
5	Achilles tendon area	19.1	23.9	3	84
6	Big toe	6.5	7.3	1	-
7	Whole foot	16.3	18.2	2	90
Mean		12.99 ± 5.13	15.99 ± 6.49^*^	2.0 ± 0.8	89.3 ± 4.1

*2-PD* two-point discrimination, *VAS* Visual Analogue Scale/Score, *TAM* total active motion, the percentage of movement on the affected side relative to the healthy side

*p = 0.015

## Discussion

ADM has long been used as soft tissue replacement therapy in the field of wound healing and tissue repair and reconstruction [17]. Commonly, the use of ADM to repair wounds has been combined with skin grafting or negative-pressure wound therapy (NPWT) [14, 18]. A handful of studies have reported regeneration of full-thickness skin defects using ADM, without skin graft or flap surgery [19–21]. As a clinical challenge, chronic vascular ulcers need to be treated by various surgical methods [22]. However, ADM also has advantages in the treatment of chronic vascular ulcers and can improve the healing rate and wound closure rate [23]. Pelanc, a double-layer structure of ADM, is generally used as a pretreatment for skin grafting, can improve the survival rate of skin grafting and accelerate wound healing [24]. We treated a case of hand trauma with a skin defect on the palm. However, the patient did not take the doctor's advice to undergo a second skin grafting surgery. At his next outpatient appointment approximately 50 days later, we found that the wound had healed. Based on this case, it is possible that ADM can not only serve as an anatomical dressing but also has the ability to stimulate wound repair. The possible mechanism is that ADM, which is similar to the dermal reticular structure, reduces the accumulation of collagen, induces angiogenesis and remodels the structure and function of the dermis [25].

In this article, we show that ADM can replace skin grafts and flaps to promote the regeneration of full-thickness skin defects. Although contraction of the wound cannot be completely avoided due to the poor extensibility of the skin at these injured sites, it is still possible that the wound can healed by regeneration. Furthermore, we found that PELNAC has certain anti-infective ability. Although the wound initially appears to resemble skin necrosis or pus formation after being covered with PELNAC for a period of time, it then starts to become drier, and the Pelnac eventually promotes regeneration. This is consistent with the results of our current animal experiments that showed that the infection rate of the wound was reduced after the use of ADM.

Compared with skin grafts and flaps, ADM presents some distinct advantages. First, patients treated with ADM are unconstrained by donor supply or damage. None of the patients needed skin or flaps harvested from other parts of the body. Second, the quality of the wounds treated with ADM was better than that of the skin or flaps in the surgical arm of the study. The comparison between affected and healthy limbs indicated comparable passive (or active) motion of the lower limb joints. In addition, the regenerated skin healed and demonstrated a satisfactory appearance and feel. After 1 year, according to the two-point discrimination measurements, the recovery with Pelnac was better than that with skin grafting [26].

However, there are shortcomings in the application of ADM to treat trauma, such as the long treatment time, which often takes several months and is a challenge for patients’ patience and trauma care. Specimens were not sampled from healed wounds, thereby limiting further pathological analysis. However, previous work conducted in our laboratory found concomitant hair regeneration for a few wounds treated with ADM [20]. These rat studies showed the infiltration of stem cells following treatment, but further work is required to determine a detailed mechanism of action.

## Conclusion

In conclusion, for select patients, 1-stage Pelnac reconstruction can be considered a novel method for inducing regrowth of the epidermis, thus warranting further research.

## Data Availability

The datasets used and/or analyzed during the current study are available from the corresponding author on reasonable request.
